# Long Term Follow-Up in Inferior Alveolar Nerve Transposition: Our Experience

**DOI:** 10.1155/2014/170602

**Published:** 2014-05-13

**Authors:** Giulio Gasparini, Roberto Boniello, Gianmarco Saponaro, Tito Matteo Marianetti, Enrico Foresta, Andrea Torroni, Giuliana Longo, Camillo Azzuni, Daniele Cervelli, Sandro Pelo

**Affiliations:** Maxillofacial Surgery Unit, Complesso Integrato Columbus, Università Cattolica del Sacro Cuore, Via Giuseppe Moscati 31, 00168 Rome, Italy

## Abstract

*Introduction*. Inferior alveolar nerve transposition (IANT) is a surgical technique used in implantoprosthetic rehabilitation of the atrophic lower jaw which has not been well embraced because of the high risk of damage to the inferior alveolar nerve (IAN). There are cases in which this method is essential to obtain good morphologic and functional rebalancing of the jaw. In this paper, the authors present their experience with IANT, analyzing the various situations in which IANT is the only surgical preprosthetic option. *Methods*. Between 2003 and 2011, 35 patients underwent surgical IANT at our center. Thermal and physical sensitivity were evaluated in each patient during follow-up. The follow-up ranged from 14 to 101 months. *Results and Conclusion*. Based on our experience, absolute indications of IANT are as follows: (1) class IV, V, or VI of Cawood and Howell with extrusion of the antagonist tooth and reduced prosthetic free space; (2) class V or VI of Cawood and Howell with presence of interforaminal teeth; (3) class V or VI of Cawood and Howell if patient desires fast implantoprosthetic rehabilitation with predictable outcomes; (4) class VI of Cawood and Howell when mandibular height increase with inlay grafts is advisable.

## 1. Introduction

Inferior alveolar nerve transposition (IANT) is a surgical technique first described by Alling [1] and Fitzpatrick [2] in 1977.

This method, to be used in implantoprosthetic rehabilitation of the atrophic lower jaw, has not been well embraced because of the high risk of damaging the inferior alveolar nerve (IAN).

Currently, the use of short implants, osteoregeneration methods, and new prosthetic solutions using interforaminal implants have further reduced the use of IANT. However, there are cases in which the transposition of the alveolar nerve is essential to obtain good morphologic and functional rebalancing of the jaw. In this paper, the authors present their experience with IANT, analyzing the various situations in which IANT is the only surgical preprosthetic option for implantoprosthetic rehabilitation of the mandible.

## 2. Materials and Methods

We selected all patients who underwent IANT surgery between 2003 and 2011 at our center. We excluded all patients treated in 2012 to have adequate follow-up to obtain final data on sensitivity related to the IAN. All patients were reexamined clinically to evaluate the IAN function between September and October 2012. We also made a summary of the surgical technique used. We did not consider the stability of system using implantologic criteria because this was not considered part of our study. The implants were made by different manufacturers and were positioned by different operators. No implant loss was reported by patients. The minimum follow-up time was 12 months.

To evaluate sensory function, we used the method described by Becelli et al. in 2002 [3]. For the clinical evaluation, we used a two-point-discrimination test, the application of a painful stimulus and thermic sensitivity.

For the two-point discrimination, a compass with two blunt metal tips was used to avoid causing pain. The tips were applied on the skin of the chin, starting on the right, with the same pressure on both points. The two-point discrimination was evaluated for each patient until the patient could no longer correctly discriminate the two points. The same procedure was then repeated on the left side. A discriminated distance of 5 mm or less was considered a satisfactory sensory response.

Nociception was evaluated with a needle applied to the skin of the chin. Patients could choose among three types of feeling: numb, dull ache, or sharp. Nociceptive sensitivity was judged good if the patient had an adequate response to stimulation with increasing intensity.

Thermal sensitivity was tested using ice cubes and warmed metal utensils. First, the ice cubes were positioned on the lower lip of the patients, first on the right side and then on the left side. The patients were asked to report the degree of cold sensitivity they experienced using the following levels: high grade, medium grade, low grade, or no sensitivity. Five minutes later, a rounded metal tool was warmed using a flame and chilled to 38°C; temperature was checked using a thermometer. Then, the heated instrument was applied first on the right side and then on the left side. Patients were asked to report their sensitivity to heat using the same rating system described above for cold sensitivity. In our analysis, hypoesthesia was defined as an imperfect or incomplete response observed during the test, paresthesia was defined as a qualitative alteration characterized by numbness or altered sensation, and anesthesia was defined as the complete loss of sensitivity. The function of the IAN was assessed by the response to the three tests.

**Figure 1:**
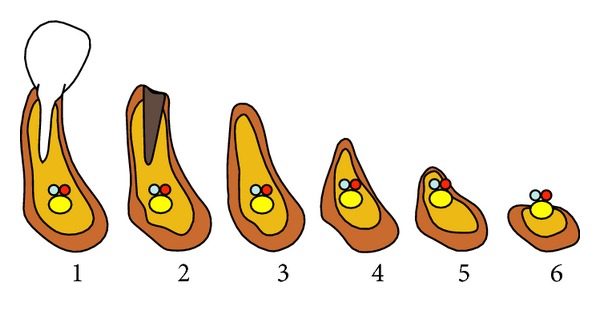
Classification system of six atrophy stages in the mandible according Cawood and Howell (1988). Atrophy stage 1: before extraction, stage 2: after extraction, stage 3: high well-rounded ridge, stage 4: knife-edge shaped ridge, stage 5: low well-rounded ridge, and stage 6: depressed bone level.

For all patients, we also considered the side and degree of the atrophy and the type of surgical instrumentation used. For atrophy assessment, we used the classification described by Cawood and Howell in 1988 [4] (Figure 1). In the literature, two types of instruments have been proposed to approach the IAN: rotary burrs (RB) and piezosurgery (PZ). In our study, both types of instruments were used, and we reported all adverse events related to the type of instrument used.

## 3. Surgical Technique

**Figure 2:**
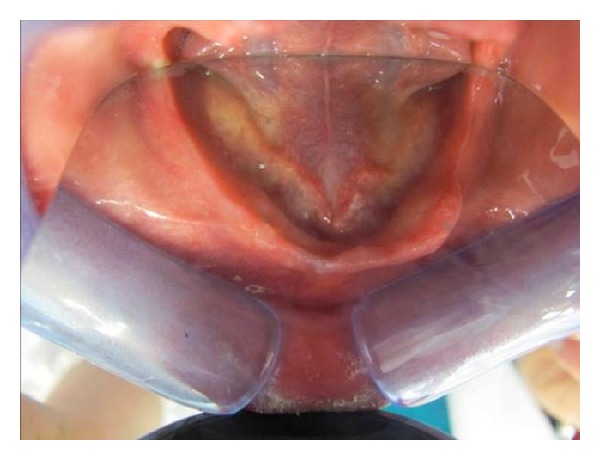
Image of the edentulous lower jaw.

**Figure 3:**
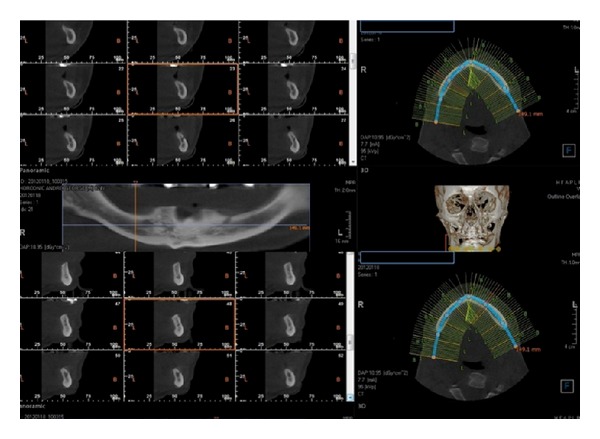
Preoperative CT dentascan imaging.

**Figure 4:**
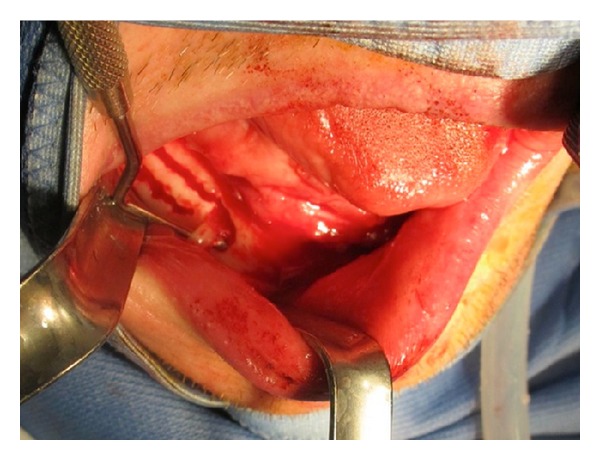
Intraoperative view: opening of the anterior operculum with the identification of the alveolar nerve at the chin foramen.

**Figure 5:**
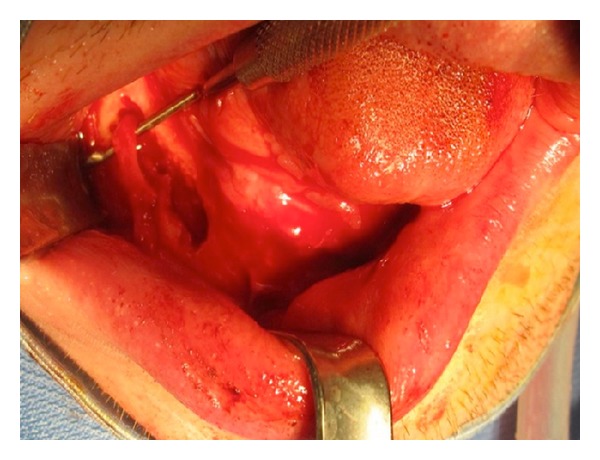
Intraoperative view: the alveolar nerve is followed along all its length.

**Figure 6:**
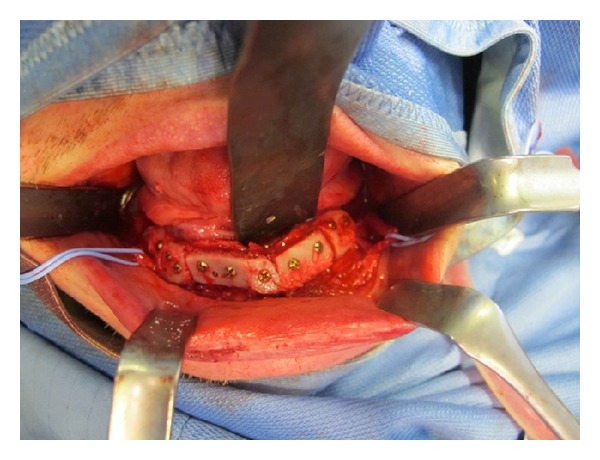
Intraoperative view: onlay grafts are positioned.

**Figure 7:**
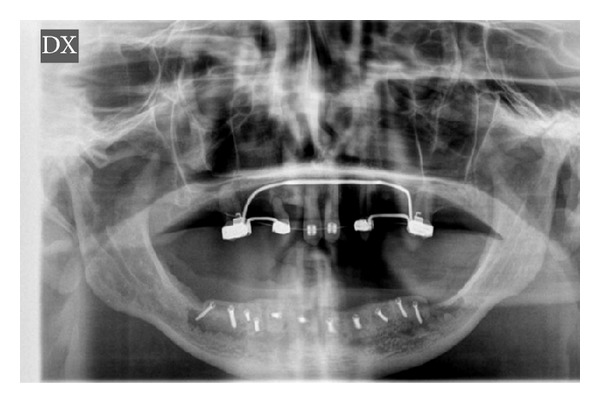
Postoperative panorex.

**Figure 8:**
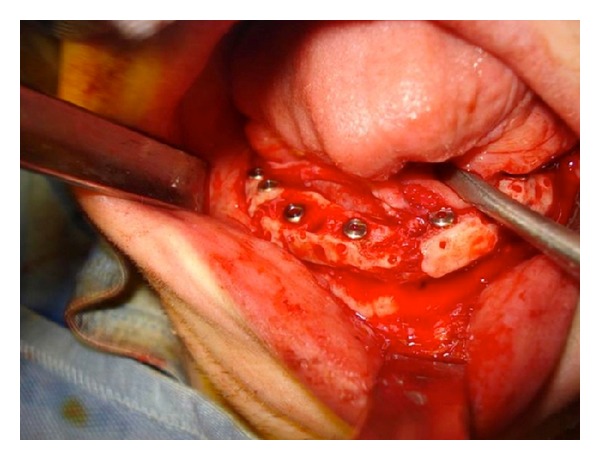
Intraoperative view: positioning of the fixtures.

**Figure 9:**
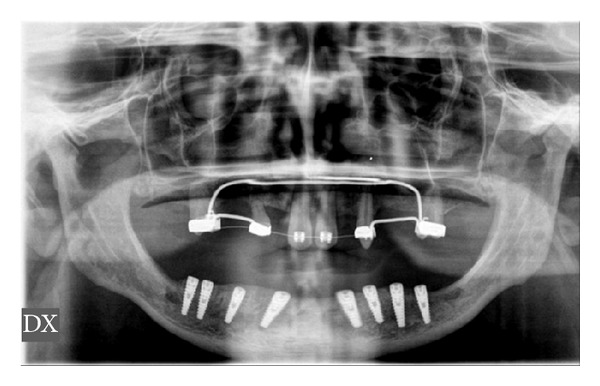
End of treatment panorex with fixtures in place.

Before beginning the surgical procedure (Figure 2), accurate studies were performed using CT dentascan (Figure 3) to evaluate the position of the loop of the alveolar nerve in relation to the mental foramen [5, 6]. We preferred to use the surgical technique described by Rosenquist in 1992 [7] and modified in 1993 by Smiler [8] which takes into account intraoperative magnification with 4x lenses. However, we made our own modifications to the technique. We draw the bone flap with a RB of small size or a PZ, taking care to mark the front portion 2 mm posterior to the mental foramen. We defined the height of the flap, respecting the vestibular cortical bone and the relationship that exists between the chin foramen, loop of IAN, and course of the mandibular canal, as observed in CT scan (Figure 4). Generally, we designed a bone flap of up to 5 or 8 mm in height and maximum 1 cm in length. This bone flap's dimensions allowed adequate intraoperative vision. We elevated the bone flap using a curved Lambotte or Freer chisel, giving gentle taps or using them as levers. With the PZ, we opened the mental foramen, dissecting the chin branch. We worked the PZ along the walls of the alveolar canal to free the length of the nerve (Figure 5). Once we extracted the nerve up to about 3 mm from the rear edge, we designed the second bone flap centred to the mandibular canal. We opened the flap with a chisel and, with the aid of a PZ, we continued to follow the IAN in its course. If necessary, we repeated the operation more posteriorly. Previously, when fixtures were required intraoperatively, we inserted them in a single surgery. Current flowcharts no longer give importance to bicorticalism to avoid the development of low resistance areas in the jaw that could create structural integrity problems [9, 10]. After inserting the fixtures, we used autologous and/or heterologous bone grafts to cover the site of the bone flap, and then we covered it with a resorbable membrane. Usually, we positioned the nerve over the membrane, although more recently we preferred to lay it under the buccal periosteum. When bone grafts were needed, they were positioned immediately after the alveolar nerve transposition (Figure 6 and Figure 7). Fixtures could also be inserted as a second-step surgery after complete integration of the bone grafts (Figures 8 and 9).

## 4. Results

Between 2003 and 2011, 35 patients underwent surgical transposition of the inferior alveolar nerve, including 19 females and 16 males ranging from 23 to 78 years of age, with an average age of 55.8. The follow-up time period ranged from 14 to 101 months, with an average of 54.2 months. In total, we performed 49 IANTs; 26 on the right side and 23 on the left (Table 1).

We performed the procedure for the correction of 33 class VI patients, 14 class V patients, and 2 class IV patients, according to the classification system of Cawood and Howell.

Complications were reported in 6 cases as follows: 1 (2.8%) case of transient anesthesia and 5 (14.3%) cases of transient hypoesthesia (spontaneously resolving after 6 months from the surgical procedure). No paresthesia was documented. Among the cases of hypoesthesia, 4 were of the discriminative type and 1 was of the thermal type with a greater impairment in recognizing cold. We decided not to make patients undergo ENG since remission of the symptoms was achieved after 6 months from the operation. Regarding instrumentation, we used RB in 16 patients with 4 (25% of the RB patients) adverse events occurring. We used PZ in 19 patients with 2 (10.5% of the PZ patients) adverse events occurring. The difference in the incidence of complications in the RB and in the PZ groups was found to not to be statistically significative (*P* = 0.3791).

Incidence of complications did not appear to be connected to Cawood and Howell class belonging (0.7337) and no significant difference according to age (0.7096) and sex was recorded (*P* = 0.3791).

Statistical analysis was performed with Fisher exact test.

## 5. Discussion

The IANT is not currently considered a safe method, and for that reason, it has received little consideration as surgical technique for preprosthetic preparation of the atrophic alveolar ridges in edentulous patients. Nevertheless, some authors continued to analyze the validity of this surgical technique, especially evaluating the residual functionality of the IAN following IANT [11–14]. The reported risk of damage to the IAN ranges between 33% and 87%; however, our results only documented a 2.8% risk of anesthesia and a 13.4% risk of hypoesthesia [11, 15, 16]. Certainly, IANT is associated with more risk than other jaw preparation techniques for implantoprosthetic rehabilitation, but in some cases IANT is the only method allowing an implant-prosthetic rehabilitation with better outcomes predictability and low biological cost for the patient. Reconstructive methods and implantoprosthetic strategies for the edentulous mandibula in Cawood and Howell classes V and VI are different, including short implants, regenerative techniques, autologous, homologous, or heterologous inlay or onlay bone grafts, and osteodistraction. However, each of these methods is connected with a certain amount of risks.

The short implants have a success rate higher than 90% after a 5-year follow-up [11, 17–19]. The problem involves the crown to implant ratio that makes the lever very unfavorable and is responsible for 45% of total stress on the bone cortex [20]. IANT would limit this problem by allowing the insertion of longer implants, but the risk of injuring the IAN makes this method unadvisable if the possibility of inserting short implants exists.

Atrophic jaw regenerative techniques, especially for what concerns vertical dimensions, have a slightly lower success rate than IANT, depending on the reference source and the method and material used, and results are not predictable. Although contextual insertion of implants is described in the literature, such implants are not possible in Cawood and Howell classes V and VI because not enough bone exists to give satisfactory stability to the fixtures. In such cases, implant surgery should be postponed [21–23].

Inlay bone graft techniques have been successfully used in many cases, but this technique is difficult to use for class V cases and almost impossible for class VI cases because the bone cannot be cut without damaging the IAN. This method always requires a second surgery for implant placement. The outcomes of onlay graft methods are not predictable, and the success rate is very low. These methods also require a second surgery for fixtures insertion [24, 25].

Distraction osteogenesis received much attention in the early 2000s but more recently has been gradually abandoned due to its intrinsic unpredictability. Distraction osteogenesis cannot be used in Cawood and Howell class VI cases for the same reasons explained above [26–29].

If an IANT is performed, fixtures can be inserted as a single-step surgery. The implant survival after IANT is good and achieved in up to 90% of the studies considered.

The survival and success rates of implants in the native jawbone after IANT are much higher than the success rate after grafts because the posterior segments of the atrophic mandible lack sufficient numbers of osteogenic cells and microvessels, making it difficult for the grafts to survive [30]. As for our results IANT has shown to be effective method for the rehabilitation of Cawood and Howell class IV, V, or VI in highly motivated patients who desire a predictable outcome in the shortest time possible [31–34]; it has not shown a high complication rate (2.8%) case of anesthesia and 5 (14.3%) cases of transient hypoesthesia, compared to the aforementioned techniques.

## 6. Conclusion

Although IANT has significant risks, including loss of nerve function, there are some clinical conditions in which it remains the only possible method for implantoprosthetic rehabilitation of the jaw.

According to our experience, if IANT is performed with the correct precautions and the appropriate instruments it is a safe procedure that generates very good results with minimum risks of injuring the neurovascular function.

In our study it has shown little amount of complications, similar to those connected with other techniques considered to be “safer.”

It is our opinion that IANT should be considered in the following cases:

1. class IV, V, or VI of Cawood and Howell with extrusion of the antagonist tooth and reduced prosthetic free space;
2. class V or VI of Cawood and Howell with presence of interforaminal teeth (patients not candidates for interforaminal implantoprosthetic methods);
3. class V or VI of Cawood and Howell if patient desires a fast implantoprosthetic rehabilitation with predictable outcomes.

To our knowledge this is the only paper in which PZ and RB are compared; both of these techniques have advantages and disadvantages but it seems that the use of both these techniques lowers the amount of complicances and speeds the surgical procedure.

## Figures and Tables

**Table 1:** Characteristics of our study sample.

	Name	Age	Sex	Cawood-Howell classification		IANT		Anesthesia		Hypoaesthesia						Paresthesia		Surgical instrument		Follow-up (months)
				DX	SN	DX	SN	DX	SN	DX			SN			Dx	Sn	PZ	RB
										D	T	N	D	T	N				
1	AS	43	F	VI	VI	X	X											X		25
2	FC	54	M	V		X													X	85
3	DF	24	M		VI		X						X						X	71
4	GS	67	F		VI		X											X	X	28
5	DD	56	M	V		X													X	94
6	SC	65	F	VI	VI	X	X			X								X		38
7	QK	32	M		VI		X											X		45
8	FF	56	F	V		X													X	96
9	H HF	23	F	V	VI	X	X											X	X	14
10	PL	64	F	VI		X												X		16
11	TR	33	M	IV		X												X		22
12	DR	78	M		V		X											X		33
13	UI	65	F	VI	V	X	X											X		50
14	OF	64	F	IV	V	X	X											X		16
15	TO	39	F	VI		X													X	73
16	ES	43	M		V		X		X										X	82
17	PI	54	M	VI		X												X		68
18	TE	61	M	VI	V	X	X											X		25
19	SA	48	F	VI		X												X		20
20	ES	65	F	VI	VI	X	X			X									X	26
21	RT	36	M		VI		X												X	71
22	FD	54	M	VI		X													X	66
23	OR	69	F	V		X													X	58
24	SA	71	F	V	VI	X	X											X		72
25	PL	73	M		VI		X						X						X	59
26	IL	66	F	VI		X													X	62
27	FR	68	M	VI	VI	X	X											X		53
28	SL	67	F	V	VI	X	X												X	51
29	AG	74	F	VI	VI	X	X												X	101
30	AS	46	F		VI		X											X		85
31	FO	65	F	VI	V	X	X											X		19
32	F BT	39	M	VI		X													X	63
33	DM	73	M	VI	VI	X	X							X				X		73
34	NB	42	F	VI		X													X	49
35	GG	76	M	V	VI	X	X											X		68

## Abbreviations

IAN: Inferior alveolar nerve
IANT: Inferior alveolar nerve transposition
RB: Rotary burr
PZ: Piezosurgery.
